# A Clinical Overview of Acute and Chronic Pancreatitis: The Medical and Surgical Management

**DOI:** 10.7759/cureus.19764

**Published:** 2021-11-20

**Authors:** Hamza Ashraf, John Paul Colombo, Vincent Marcucci, Jonathan Rhoton, Oluwatofunmi Olowoyo

**Affiliations:** 1 Medical Education, St. Peter's University Hospital, New Brunswick, USA; 2 Medical Research, St. Peter's University Hospital, New Brunswick, USA; 3 Medical Research, St. George's University School of Medicine, True Blue, GRD; 4 Surgery, Jersey Shore University Medical Center, Neptune, USA; 5 Surgery, Hackensack University Medical Center, Hackensack, USA; 6 Internal Medicine, St. Peter's University Hospital, New Brunswick, USA

**Keywords:** pancreatitis, surgery, surgical management of pancreatitis, pancreas disease, pancreatitis causes

## Abstract

An inflammatory process involving the pancreas, known as pancreatitis, can be categorized as either acute or chronic and may present in one of many ways. The clinical manifestations of acute pancreatitis are generally limited to epigastric or right upper quadrant pain, while manifestations of chronic pancreatitis are broader and may include abdominal pain in tandem with signs and symptoms of pancreatic endocrine and exocrine insufficiency. An understanding of the initial insult, proper classification, and prognosis are all factors that are of paramount importance as it pertains to managing patients who are afflicted with this disease. Our review delves into the depths of pancreatitis by exploring the embryology and anatomy of the pancreas, the pathophysiology and etiology of acute and chronic pancreatitis, and the medical and surgical management of acute and chronic pancreatitis.

## Introduction and background

Pancreatitis is a pathology with many underlying facets. Understanding pancreatitis requires a grasp of anatomical, physiological, and pathological elements, while also requiring an appreciation for its two broad forms of existence and its many forms of management. Although medical management tends to be the initial stay of treatment for uncomplicated pancreatitis, there are still indications for surgical management. As time has progressed, newfound literature and studies have led to advancements and innovations that have changed the direction of surgical treatment, steering away from open surgical procedures, and more toward minimally invasive procedures that yield similar outcomes while still maintaining a strong level of efficacy.

## Review

Pancreatic embryology

The development of the human pancreas begins approximately on gestational day 26. It begins as three endodermal buds on the caudal portion of the foregut. The dorsal bud gives rise to the majority of pancreatic tissues, namely, the upper portion of the head, isthmus, and tail. The right ventral bud develops into the inferior portion of the head. In most cases, the left ventral bud will gradually regress, if it does not, it can lead to the congenital malformation known as an annular pancreas [1]. As the stomach, duodenum, and ventral mesentery begin to rotate, the pancreas will come to lie in the retroperitoneal space [1,2]. Early in the fetal period, the secretory portions of the pancreas, known as acini, begin to form around the forming ducts of the parenchyma and hormone secretion begins around week 10 of gestation [1,3].

Pancreatic gross anatomy

The pancreas is a grossly appearing lobulated abdominal organ with both exocrine and endocrine functions. Its main functions are to secrete the hormones insulin, glucagon, and somatostatin, as well as synthesize digestive enzymes. The pancreas is an intra-abdominal organ that traverses the right and left upper quadrants. It is divided into four regions: the head, neck, body, and tail. The pancreatic head lies in the right upper quadrant off the midline, adjacent to the descending duodenum with the tail extending into the hilum of the spleen in the left upper quadrant. It lies posterior to the stomach and anterior to the abdominal aorta and inferior vena cava [4,5]. The pancreas is primarily a retroperitoneal organ with the tail considered to be intraperitoneal as it is located within the splenorenal ligament [4].

The main pancreatic exocrine secretions drain through a series of smaller ducts that begin in the tail. These ducts empty into the main pancreatic duct, which runs near the posterior surface of the body and neck. Within the pancreatic head, the main pancreatic duct lies immediately to the left of the common bile duct (CBD) draining from the liver and gallbladder. The CBD and main pancreatic ducts penetrate the descending duodenal wall together at the hepatopancreatic ampulla of Vater, which is surrounded by the sphincter of Oddi [4,6,7]. Further drainage of the pancreatic head is accomplished via the accessory pancreatic duct [4]. The accessory pancreatic duct passes from the superior portion of the pancreatic head and opens into the duodenum via the minor papilla, an opening that is typically located approximately 2 cm proximal to the opening of the major pancreatic duct [7].

The blood supply to the pancreas is achieved through branches of the celiac trunk and superior mesenteric artery (SMA). Blood to a large portion of the pancreas is supplied through the pancreatic branches of the splenic artery, a branch of the celiac trunk. The head of the pancreas has additional blood supply from the superior and inferior pancreaticoduodenal arteries, which are branches of the gastroduodenal (a terminal branch of the common hepatic artery) and the SMA, respectively [1,6]. Venous drainage is to the hepatic portal system and occurs via the splenic vein and superior mesenteric vein (SMV). The splenic vein receives venous tributaries from the tail and body of the pancreas, while the head and neck of the pancreas drain into the superior and inferior pancreaticoduodenal veins. The superior veins drain partly into the right gastroepiploic vein and partly into the portal vein directly. The inferior pancreaticoduodenal veins drain into the SMV [6]. Lymphatic drainage of the pancreas follows the pancreatic arteries. The body and tail of the pancreas drain into the retropancreatic nodes. The superior half of the head and neck drains into the celiac lymph nodes and the inferior half of the head drains into the superior mesenteric nodes [4-6].

Pancreatic microscopic anatomy

The pancreas is surrounded by a thin layer of loose connective tissue, which forms a capsule surrounding the gland. This capsule then penetrates the gland as various septa, giving the gland its lobulated gross appearance [8]. Each lobule is composed of various serous secretory units, known as acini. The acini form the exocrine portion of the pancreas and empty their secretions via the pancreatic ducts. Each acinus is made up of clusters of simple epithelia composed of pyramidal serous cells, with their apices surrounding a central duct lumen, lined with low simple cuboidal epithelium [8]. As the ducts gradually increase in size, this low cuboidal epithelium is replaced with stratified cuboidal epithelium [9].

Dispersed between the acini are the islets of Langerhans, which are responsible for the endocrine functions of the pancreas. The islets are composed of three main cell types, alpha, beta, and delta cells, which secrete glucagon, insulin, and somatostatin, respectively [6,8,9]. The islet cells have a polygonal histological orientation and are arranged in short, irregular cords that are intertwined within a network of fenestrated capillaries [8]. Islets vary in size and are found in higher concentrations in the pancreatic tail [8]. Additional endocrine cell types found in the islets are responsible for the secretion of other hormones such as vasoactive intestinal peptide (VIP), pancreatic polypeptide, motilin, serotonin, and substance P [9]. Adipocytes can be found within the pancreatic parenchyma in increasing amounts in elderly individuals, corresponding with age-related atrophy of the gland [9].

Pathophysiology of acute pancreatitis

Acute pancreatitis (AP) is a common clinical condition resulting from an acute injury to the pancreas usually causing self-limiting pancreatic inflammation [10]. A severe multi-system inflammatory response can occur in up to 25% of patients diagnosed with pancreatitis, in which 30% to 50% will expire [10]. There are multiple etiologies responsible for AP, with the two most common being gallstones, which account for up to 40% of cases, and alcohol, which is responsible for approximately 30% of cases [11]. Other causes of AP include the following: medications such as angiotensin-converting enzyme (ACE) inhibitors, sulfa-based drugs, furosemide, azathioprine, 6-mercaptopurine, and valproate; infections such as coxsackievirus B, cytomegalovirus, and hepatitis A and E; inherited mutations in cationic trypsinogen (PRSS1) or cystic fibrosis; mechanical etiologies such endoscopic retrograde cholangiopancreatography (ERCP), abdominal trauma, pancreatic cancer, sphincter of Oddi stenosis, and pancreatic divisum; and metabolic causes such as hypertriglyceridemia and hypercalcemia [10,11].

The pathophysiology of gallstone pancreatitis is a result of mechanical obstruction of the ampulla from a stone or edema caused by the passage of the stone through the duct inducing pancreatic ductal hypertension and acinar cellular injury [11]. The metabolization of alcohol into toxic metabolites increases enzymatic content and destabilizes lysosomal and zymogen granules, with sustained increases in calcium overload, and activated pancreatic stellate cells potentiate acinar cell autodigestion and cell death [10]. Irrespective of the inciting injury, AP is a consequence of acinar cell disruption and enzymatic release triggering intra-acinar zymogen activation and cellular autodigestion [11]. The effector enzymes trypsin, chymotrypsin, elastase, phospholipase A2, and lipase break down tissue membranes, causing apoptosis, necrosis, edema, vascular damage, hemorrhage, and a subsequent localized and systemic inflammatory response [10,11].

The severity of AP is thought to be correlated with the degree of necrosis related to apoptosis [11]. A higher necrosis-to-apoptosis ratio correlates to the increasing severity of illness [11]. Bhatia et al. outline the current understanding of cellular mechanisms involved in AP [10]. During the pathologic process, activated enzymes and cytokines may enter the peritoneal cavity causing a chemical burn and third spacing of fluid leading to peritonitis and ascites [11,12]. Enzymes and cytokines may also enter the systemic circulation and cause a systemic inflammatory response that can result in acute respiratory distress syndrome (ARDS) and acute kidney injury [11,12]. Phospholipase A2 is theorized to injure alveolar membranes in the lungs, which are directly associated with ARDS [11,12]. The systemic complications are mainly due to increased capillary permeability and decreased vascular tone, which results from released cytokines and chemokines, particularly interleukin (IL)-1, IL-6, IL-8, monocyte chemoattractant protein-1, and macrophage inflammatory protein-1, causing direct epithelial and obstructive damage within the pancreas [11-13]. AP also increases the risk of infection and sepsis by compromising the gastrointestinal barrier, leading to bacterial translocation from the gut lumen into circulation [11].

The pathophysiology of AP occurs in two phases: an early and a late phase. The early phase encompasses the first week of disease manifesting in the systemic inflammatory response [12]. In this phase, clinical severity and treatment are based upon type and degree of organ failure [13]. The late phase persists beyond the one-week mark and may last for weeks to months. It is more likely to occur in severe forms of disease and is marked by persistent organ dysfunction and local complications [12,13]. Treatment in the late phase relies heavily on a set of morphologic criteria determined by radiographic features [12].

AP can be classified based upon the severity of illness and subtypes, which are determined by the Revised Atlanta Classification. The severity of AP is related to local pancreatic or peripancreatic complications and systemic effects, such as transient or persistent organ failure [11,13,14]. Local complications include pancreatic and/or peripancreatic necrosis, fluid collections, splenic vein thrombosis, pseudoaneurysm formation, and gastric outlet dysfunction [11-14]. Systemic complications result in organ and multi-system organ compromise that is based upon the modified Marshall scoring system for organ dysfunction. There are three degrees of severity based on within the classification: mild, moderately severe, and severe. To be classified as mild, there should be no evidence of organ dysfunction and no local or systemic complications [13]. The moderately severe disease must include organ dysfunction that resolves in less than 48 hours (transient organ failure) and/or local or systemic complications without persistent organ failure (lasting longer than 48 hours) [13,14]. For severe AP, there must be evidence of persistent organ failure of single or multiple organs [14]. The Atlanta Classification further divides AP into two subtypes based on contrast-enhanced computed tomography (CECT) imaging: interstitial edematous pancreatitis (IEP), which is the most common, and necrotizing pancreatitis (NP), which is further subdivided into parenchymal, peripancreatic, or combined, of which the latter is more common [12,14]. NP may be sterile or infected with the gas formation on imaging as the main component to suggest an infectious process [13,14]. Each subtype of AP can be complicated by a fluid collection that may be encapsulated or unencapsulated. Fluid collections tend to be unencapsulated if the onset is less than four weeks [13]. These collections include acute peripancreatic fluid collection (APFC) in IEP and acute necrotic collection (ANC) in NP [14]. Fluid collections that are encapsulated usually occur after four weeks, and commonly include pseudocyst formation in IEP and walled-off necrosis (WON) in NP [11,14].

Diagnosis of acute pancreatitis

The diagnostic algorithm for AP encompasses laboratory markers and radiographic imaging to support the clinical presentation of a patient presenting with severe epigastric abdominal pain with or without radiation, who has a history of alcohol use or gallstones [11]. The diagnosis can be made if at least two of the following criteria are met: abdominal pain consistent with the disease process, serum amylase and/or lipase greater than three times the upper limit of normal, and characteristic findings on CECT [11]. Serum pancreatic enzyme levels peak on the first day and normalize around three to seven days, although lipase has greater sensitivity and specificity than amylase both early and later in the disease course [11,15,16]. Urine trypsinogen-2 will also help support the diagnosis as the sensitivity and specificity are greater than 90% for AP [11]. Other supporting laboratory values are elevated white blood cell (WBC) count, hematocrit, and blood urea nitrogen (BUN) due to the third-spacing of fluids [11,12,14,16]. Hyperglycemia may result from pancreatic insufficiency and hypocalcemia due to saponification of peripancreatic fatty tissue [11].

Prognosis of acute pancreatitis

The prognostic indicators for AP are largely an estimate from a set of criteria set forth by several scoring systems including Ranson criteria, Glasgow prognostic criteria, Acute Physiology and Chronic Health Evaluation II (APACHE II) classification system, and Balthazar CT-enhanced scoring system, among others [11,17]. The bedside index for severity in acute pancreatitis (BISAP) score is a newer scoring system that can be used to quickly assess the patient’s mortality risk using fewer parameters than the Ranson criteria [11,17]. The initial risk assessment should include the factors that have a high predictability of a severe course. These factors include age greater than 60 years, comorbid health problems, BMI greater than 30, chronic alcohol use, presence of systemic inflammatory response syndrome (SIRS), laboratory markers of hypovolemia (e.g., elevated BUN and hematocrit), and pleural effusions and/or infiltrates on chest X-ray [11]. The Ranson criteria contain 11 parameters used to assess the severity of alcoholic pancreatitis and the modified Ranson criteria contain 10 parameters used to assess gallstone pancreatitis. For alcoholic pancreatitis, five parameters are assessed on admission: age greater than 55 years, WBC count greater than 16,000 cells/cm^2^, blood glucose greater than 200 mg/dL (11 mmol/L), serum aspartate aminotransferase (AST) greater than 250 IU/L, and serum lactate dehydrogenase (LDH) greater than 350 IU/L [17]. At 48 hours, another six parameters are evaluated, including a serum calcium less than 8.0 mg/dL (less than 2.0 mmol/L), a decrease in hematocrit greater than 10% from baseline, partial pressure of oxygen (PaO2) less than 60 mmHg, a BUN increase by 5 mg/dL or more (1.79 mmol/L or more) despite intravenous (IV) fluid hydration, base deficit greater than 4 mEq/L, and fluids need greater than 6 L [17].

For gallstone pancreatitis, the modified Ranson criteria require five initial parameters on admission, which are age greater than 70 years, WBC greater than 18,000 cells/cm^2^, blood glucose greater than 220 mg/dL (greater than 12.2 mmol/L), serum AST greater than 250 IU/L, and serum LDH greater than 400 IU/L [17]. At 48 hours, additional five parameters are taken including serum calcium less than 8.0 mg/dL (less than 2.0 mmol/L), a decrease in hematocrit greater than 10%, BUN increased by 2 or more mg/dL (0.7 or more mmol/L) despite IV fluid hydration, base deficit greater than 5 mEq/L, and fluids need greater than 4 L [17]. The scoring system is as follows: 0 to 2 points correlate to a mortality risk of 0% to 3%, 3 to 4 points correlate to 15%, 5 to 6 points correlate to 40%, and 7 to 11 points correlate to nearly 100% mortality risk [17]. A comparative study done in 2021 discussed some of the limitations of the Ranson criteria when comparing it to the APACHE II system and BISAP score. It was determined that the APACHE II system was more sensitive than the Ranson criteria and the BISAP score was more specific than the Ranson criteria [17]. In addition to its lack of convenience, the Ranson criteria disregard the BUN levels in the prognostic algorithm until the 48-hour mark, which is associated with increased severity of AP and/or increased mortality [17]. It has been stipulated that elevated BUN reflects intravascular volume depletion, which may be associated with inflammatory mediators in response to acute inflammation. The same train of thought can be used for explaining the rise in hematocrit seen in severe cases of AP, which the Ranson criteria fail to take into consideration until the 48-hour mark [17]. Hematocrit greater than 47% on admission has been determined to be a sensitive predictor of pancreatic necrosis during admissions [17]. Other markers used to stage AP include levels of C-reactive protein (CRP) and interleukin-6 (IL-6), with increased levels corresponding to increased severity [17]. An increase in CRP/albumin ratio has shown to be reliable in categorizing more severe diseases [18]. Many other biological markers have shown promise in predicting the severity of AP (e.g., trypsinogen activation peptide, phospholipase A2, and polymorphonuclear elastase); however, further research is needed to accurately assess their reliability in the prognostic algorithm [17]. Table 1 summarizes the Ranson criteria, BISAP, and APACHE II criteria.

**Table 1:** Comparing and contrasting the prognostic scores/criteria for acute pancreatitis. BISAP = bedside index for severity in acute pancreatitis; APACHE = Acute Physiology and Chronic Health Evaluation; WBC = white blood cell; AST = aspartate aminotransferase; LDH = lactate dehydrogenase; BUN = blood urea nitrogen; SIRS = systemic inflammatory response syndrome; MAP = mean arterial pressure; HR = heart rate; RR = respiratory rate; A-a DO2 = alveolar to arterial difference of oxygen; FiO2 = fraction of inspired oxygen; Hct = hematocrit; PaO2 = partial pressure of oxygen.

Ranson criteria (on admission)	BISAP (first 24 hours of admission)	APACHE II (acute physiology score)
WBC > 16,000	BUN > 25 mg/dL	Temperature < 36°C or ≥ 38.5°C
Age > 55	Age > 60	MAP < 70 or ≥ 110
Glucose > 200 mg/dL	≥ 2 SIRS criteria	HR < 70 or ≥ 110
AST > 250	Impaired mental status	RR < 12 or ≥ 25
LDH > 350	Pleural effusion present	A-a DO2 (on FiO2 of ≥ 0.5) < 200 or ≥ 200 or PaO2 (on FiO2 < 0.5) < 70 or ≥ 70
48 hours after admission		pH < 7.33 or ≥ 7.50 or if no ABG, HCO3 < 22 or ≥ 32
Hct drop > 10 %		Na < 130 or ≥ 150
BUN increase > 5 mg/dL		K < 3.5 or ≥ 5.5
Ca2+ < 8 mg/dL		Creatinine < 0.6 or ≥ 1.5
PaO2 < 60 mmHg		Hct < 30 or ≥ 46
Base deficit > 4 mg/dL		WBC < 3,000 or ≥ 15,000
Fluid needs > 6 L		Age > 44
		Chronic organ insufficiency

Pathophysiology of chronic pancreatitis

Chronic pancreatitis (CP) is persistent long-standing inflammation of the pancreas that results in permanent structural damage marked by fibrosis and ductal strictures leading to an irreversible decrease in exocrine and endocrine pancreatic function [19,20]. The reported prevalence of CP across the United States and Europe ranges from 0.2% to 0.6% and the incidence is about 7-10 per 100,000 [20]. The etiology of CP is multifactorial, although the most common inciting factors include chronic alcohol consumption, which accounts for over 50% of cases, and tobacco smoking [19]. Studies show an independent dose-response relationship between both alcohol and smoking in the development of CP and it is likely that both risk factors exhibit a synergistic effect [19-22]. However, not all patients with these risk factors develop AP or CP; suggesting other cofactors are involved. Other etiologic factors include genetic mutations in the cationic trypsinogen gene (PRSS1), serine peptidase inhibitor Kazal type 1 (SPINK1), and the cystic fibrosis transmembrane regulator (CFTR) [19,20]. Chronic obstructive causes of CP include pancreatic ductal strictures, tumor mass effect, pancreatic divisum, and sphincter of Oddi dysfunction [20]. There are several autoimmune predisposing factors including systemic Immunoglobulin G4 (IgG4) disease (type 1) and idiopathic (type 2) [19]. Tropical pancreatitis is an idiopathic cause in areas such as India, Indonesia, and Nigeria, marked by an early age of onset, large ductal calculi, and accelerated disease course [19,20]. Additional risk factors include chronic hypercalcemia and hyperlipidemia [20].

The onset of pancreatic fibrogenesis in CP is caused by injury to the interstitial mesenchymal cells, duct cells, and/or acinar cells depending on the causal factor [20]. The initial insult to pancreatic parenchymal tissue is the inciting factor associated with necrosis and/or apoptosis and subsequent release of cytokines and growth factors such as tumor growth factor-alpha 1 (TGF-α1), interleukin-8 (IL-8), platelet-derived growth factor (PDGF), and chemokines from either polymorphonuclear cells, macrophages, and/or resident epithelial or mesenchymal cells [17,19,20]. Phagocytosis of necrotic debris and release of cytokines causes activation and transformation of pancreatic stellate cells (PSCs) or pancreatic resident fibroblasts into myofibroblast-like cells that proliferate and express α-smooth muscle actin (α-SMA) to secrete collagen I, III, and fibronectin [20]. The myofibroblast-like deposit newly formed extracellular matrix (ECM) and replace inflammatory infiltrate by producing matrix metalloproteinases (MMPs), which break down normal pericellular ECM [11,19]. Furthermore, the expression of transforming growth factor-beta 1 (TGF-β1) via PSCs causes autocrine inhibition of MMPs and thus reduces collagen degradation and facilitates fibrogenesis [17,20]. It has been hypothesized that ethanol consumption may be associated with activation of resident fibroblasts directly bypassing this inflammatory process and causing induction of lithogenic proteins, increasing the viscosity of pancreatic secretions, and forming protein plugs, which cause ductal obstruction and subsequent acinar cell damage and atrophy over time [11,19,20]. With each successive pancreatic insult, this pattern of fibrogenesis and atrophy ensues resulting in diminished pancreatic function. There is evidence that shows an increased risk of recurrent pancreatitis after an initial episode of AP and increased risk of CP with individuals who suffer from recurrent bouts of pancreatitis associated with alcohol abuse and tobacco smoking [21].

Chronic damage and remodeling of pancreatic parenchyma lead to exocrine and endocrine pancreatic insufficiency. When protease and lipase secretions are reduced to less than 10% of normal, the patient develops malabsorption characterized by steatorrhea malnutrition and weight loss [20]. Glucose intolerance may ensue at any time due to insulin deficiency, although overt insulin-dependent diabetes mellitus usually occurs late in the disease course [16]. Patients suffering from CP are at a significantly higher risk of developing hypoglycemia due to the resultant damage and reduction in alpha cells [16]. Additional complications associated with CP include the formation of pseudocysts, bile duct or duodenal obstruction, pancreatic duct disruption resulting in ascites or pleural effusion, splenic vein thrombosis, which can cause gastric varices, pseudoaneurysms of arteries near the pancreas or pseudocyst, and an increased risk of pancreatic adenocarcinoma with the risk being greatest in hereditary and tropical pancreatitis [16,20]. Signs and symptoms of CP include constant or intermittent epigastric abdominal pain, which is usually postprandial and relieved by sitting upright or leaning forward [20,22]. Glucose intolerance, hypoglycemia, weight loss, fatigue, abdominal distention, and steatorrhea are all classical signs of CP [22]. About 10% to 15% of patients report no pain and present only with symptoms of malabsorption [20,22].

Diagnosis of chronic pancreatitis

The diagnosis of CP relies on clinical assessment, imaging, and pancreatic function tests [20]. MRI coupled with magnetic resonance cholangiopancreatography (MRCP) is the preferred imaging modality as it can reveal pancreatic masses and provide optimal visualization of ductal abnormalities consistent with CP. The use of IV secretin during MRCP increases sensitivity for detecting ductal abnormalities and allows for a functional assessment [20]. Pancreatic function tests are useful when imaging studies are non-diagnostic. Direct tests involve IV infusion of cholecystokinin (CCK) or secretin to measure the production of digestive enzymes or bicarbonate, respectively [20]. The diagnostic accuracy is highest with these tests when conducted early in the disease course [22]. However, these interventions are invasive, time-consuming, and not well standardized [20,22]. Indirect tests involve analysis of blood or stool samples. Serum levels of trypsinogen less than 20 ng/mL are highly specific for CP. A 72-hour fecal fat test in patients on a high-fat diet is diagnostic for steatorrhea [20]. Decreased levels of fecal chymotrypsin and elastase suggest pancreatic insufficiency [20]. The indirect tests are readily available, more convenient, less invasive, and inexpensive, although they are less accurate in diagnosing the disease in its earlier stages [20].

Medical management of pancreatitis

Initial management of patients presenting with AP focuses primarily on the acute symptoms. All patients with pancreatitis are initially treated with aggressive fluid resuscitation, pain control, and temporary discontinuation of oral feeds [23]. Currently, there is no specific consensus on the number of fluids given. However, some researchers suggest giving at least 6L at a rate of 250-300 ml/H [24]. Guidelines currently recommend IV opioids under the patient's control [23]. Initially, patients are made nothing per mouth (NPO) in the early stages of pancreatitis to allow the pancreas to recover. Subsequently, patients are recommended to be placed on an oral low-fat soft diet to promote faster recovery and decrease the risk of infection [25]. Severe pancreatitis, which includes any form of organ failure such as acute kidney injury, signs of an inflammatory response, and altered mental status, requires more aggressive ICU management [24]. Antibiotics are typically used in patients suspected of having abscesses, necrosis, or extrapancreatic indications [23].

The medical management of CP involves pain control and replacement of exocrine and endocrine function. Initial history and exam must rule out other causes of pain before treatment. Analgesia is accomplished with the use of nonsteroidal anti-inflammatory drugs (NSAIDs) and acetaminophen, with opioids reserved for breakthrough or refractory pain [26]. Opioids can be given in combination with antidepressants such as selective serotonin reuptake inhibitors (SSRIs), serotonin-norepinephrine reuptake inhibitors (SNRIs), tricyclics, and gabapentin [26]. Although antioxidants are sometimes used with other drug combinations, there is a lack of sufficient evidence showing a significant effect in pain reduction [27]. Exocrine pancreatic insufficiency develops several years after the initial onset of CP. Patients typically develop symptoms of fat malabsorption such as steatorrhea, diarrhea, flatulence, and vitamin deficiency. Initial assessment includes measuring fecal elastase levels, trypsin, and BMI, and assessing for nutritional and vitamin deficiency. The maximum dose of an enteric-coated enzyme (90,000 USP) taken with each meal effectively reduces symptoms [28]. Metformin is typically preferred as first-line in patients who develop diabetes secondary to pancreatitis, as it has been shown to help reduce the risk of pancreatic cancer [29].

Surgical management of acute pancreatitis

While the role of surgery in the management of AP has become more restricted over the past two decades, there is still a collection of indications for its use, particularly those nested in the treatment of the possible sequelae of AP [30]. Intra-abdominal hypertension (IAH) is an early phenomenon of severe acute pancreatitis (SAP) that is typically caused by the inflammatory process in the pancreas coupled with aggressive fluid resuscitation [31]. The incidence of IAH in patients with SAP ranges from 60% to 80% [31]. Initial elevations in intra-abdominal pressure can suddenly peak as severe intra-abdominal inflammation, visceral edema, and capillary leakage can further contribute to the progression of a significant amount of ascites, which may trigger the transformation of IAH into abdominal compartment syndrome (ACS) [32]. ACS, a potentially lethal consequence of IAH, is defined as the combination of intra-abdominal pressure greater than 20 mmHg and new-onset organ dysfunction [30]. The initial approach to managing IAH and ACS is through medical therapy via nasogastric decompression, percutaneous drainage of the ascitic fluid, and neuromuscular blockade [31,33]. Patients who fail to respond appropriately to medical therapy require surgical decompression. Surgical decompression is accomplished primarily through a midline laparotomy extending from the xiphisternum to the pubis, as this method allows for visualization of the bowel to assess for ischemic changes [30,31]. A second surgical approach is to use a transverse incision that extends bilaterally below the costal margins to form a full-thickness laparotomy, a method more likely to lead to successful primary closure [30]. A third surgical approach is via a subcutaneous fasciotomy of the linea alba through short horizontal skin incisions [30].

Patients with AP secondary to gallstone impaction in the sphincter of Oddi are potential candidates for ERCP, a procedure done by advancing an endoscope into the second part of the duodenum and progressing it through the ampulla of Vater and pancreatobiliary tract [34,35]. Although over 70% of patients will pass the stone into the duodenum without further complications, distinguishing between patients who may have an uncomplicated course from patients who may progress to SAP is difficult [36-39]. Therefore, the decision of whether to proceed with ERCP must be made with an understanding of the clinical context of the patient’s current state of disease. ERCP has limited value in patients with mild suspected biliary pancreatitis who show signs of clinical improvement, henceforth, MRCP and endoscopic ultrasound (EUS) are better choices for diagnostic purposes [34]. If there is suspicion for ongoing biliary obstruction, such as signs and symptoms of ascending cholangitis, total bilirubin greater than 4 mg/dL, or choledocholithiasis saw on imaging, then ERCP is indicated [40]. Total bilirubin levels less than 4 mg/dL but greater than 1.8 mg/dL require additional imaging with either EUS or MRCP to assess for choledocholithiasis [40]. Furthermore, the American Gastroenterological Association (AGA) recommends that a laparoscopic cholecystectomy be performed during the admission for pancreatitis, as this decreases the risk of recurrent gallstone pancreatitis, symptomatic choledocholithiasis, and cholangitis [40,41]. In patients who are poor surgical candidates, an ERCP with biliary sphincterotomy or biliary stent placement may be an adequate alternative in the prevention of recurrent biliary events [40]. Biliary endoscopic sphincterotomy (EST) is a procedure in which there is incising of the biliary sphincter and the intraduodenal portion of the CBD using a high-frequency current applied with a sphincterotome, which is inserted into the papilla [42].

Pancreatic and peripancreatic necrosis are serious complications of AP, with secondary infection of necrotic tissue being the leading cause of death in patients afflicted [43]. Open surgical necrosectomy, which was originally the treatment of choice for managing pancreatic necrosis, has increasingly been replaced by minimally invasive modalities including endoscopic drainage and percutaneous catheter drainage [43]. The minimally invasive procedures are performed in a “step-up” approach. The step-up approach works towards neutralizing the primary source of infection, as opposed to total excision of the infected necrotic tissue [44]. The first step consists of either percutaneous or endoscopic drainage through the left retroperitoneum, which also allows for the possibility of retroperitoneal necrosectomy if warranted in the future [44]. A failure of the patient to clinically improve facilitates performing a second drainage procedure, especially if the position of the primary drain was found to be inadequate, or if additional drainable fluid collections were discovered. Continued failure to clinically improve leads to progression to the next step, which is a video-assisted retroperitoneal debridement (VARD) with a concomitant postoperative lavage [44]. It was found that the minimally invasive step-up approach reduced the rate of major complications and death in patients with NP and infected necrotic tissue as compared to open necrosectomy [44].

Endoscopic necrosectomy is universally performed in patients who have experienced an episode of SAP, typically four or more weeks after onset, which is when a necrotic fluid collection is usually formed [45]. During an endoscopic necrosectomy, an endoscope is advanced to the level of either the stomach or duodenum, at the point where the necrotic tissue is typically seen pressing against the viscera [45]. The wall of either the stomach or duodenum is then punctured to create an opening for a guidewire to be inserted and coiled inside the necrotic cavity [45]. The guidewire is used to dilate the wall of the stomach or duodenum to at least 15 mm, after which a stent is inserted across the length of the opening to the point of the necrotic cavity [45]. The necrotic pancreatic tissue is then endoscopically removed from inside the cavity and dropped into the stomach or duodenum [45]. Contrarily, open necrosectomy is performed primarily through an upper transverse subcostal laparotomy, which can provide an access point to the site of pancreatic necrosis gained through the gastrocolic ligament [46]. Removal of the necrotic segment is then done using blunt manual dissection and debridement followed by lavage with normal saline [46]. The challenges of an open necrosectomy stem from the fact that accessing the pancreas is difficult since it sits behind the stomach in the retroperitoneum [45]. Surgical necrosectomy also carries complications associated with a large abdominal incision, including ventral hernia defects, as well as the possibility of enterocutaneous fistula formation [45].

A pancreatic pseudocyst can be defined as a non-necrotic encapsulated fluid collection confined within a well-defined inflammatory wall [30]. Approximately 5% to 15% of episodes of pancreatitis are complicated by the development of pseudocysts [47]. This number is much higher in patients who experience SAP, with approximately 50% of these patients developing pseudocysts [48]. Additionally, approximately 70% of pseudocysts resolve on their own without any treatment; however, a pseudocyst complicated by infection, a symptomatic pseudocyst, and a pseudocyst greater than 6 cm are all indications for intervention [49]. Recent studies have found that percutaneous drainage of infected or symptomatic pancreatic pseudocysts is more efficacious than surgical drainage, with some studies demonstrating a mortality benefit [47-49]. However, the treatment of choice to drain a non-infected pancreatic pseudocyst is via endoscopy [47,50]. Endoscopic treatment of a pancreatic pseudocyst is aimed at creating a connection between the cavity of the pseudocyst and the lumen of the gastrointestinal tract [50]. This is accomplished via either transpapillary drainage or transmural drainage [50]. The therapy of choice is transpapillary drainage if the pseudocyst comes into direct contact with the pancreatic duct [50]. If the pseudocyst causes a visible impression on the duodenal wall, then transmural drainage becomes the treatment of choice, with exact positioning determined via a CT scan or an EUS [50]. Surgical drainage is indicated in patients with pseudocysts complicated by infection and necrosis, pseudocysts associated with either stricture or dilation of the pancreatic duct, suspected cystic neoplasia, bile duct stenosis, and perforation and hemorrhage [50]. For pseudocysts that are directly attached to the posterior wall of the stomach, a pseudo-cystogastrostomy can be performed, while cysts that are smaller than 4 cm in size and are in the head or uncinate process can be managed with pseudo-cystoduodenostomy [50].

Surgical management of chronic pancreatitis

Approximately 40% to 75% of patients suffering from CP will require surgery at some point during the course of their disease to alleviate symptoms of pain [51]. The Puestow procedure, also known as a longitudinal pancreaticojejunostomy, is the treatment of choice in patients with CP, a dilated pancreatic duct, no inflammatory mass, and abstinence from alcohol for more than one year [51]. This surgical procedure is done via the creation of an opening along the anterior surface of the pancreas from the head extending as far into the tail as possible, after which, calcified stones are removed and a Roux-en-Y jejunostomy is attached to the sides of the pancreas [51]. Traditionally, the Puestow procedure was done through an open incision; however, recent advancements have made it possible to perform this procedure through laparoscopic and robotic techniques [52]. The procedure is associated with a morbidity and mortality rate of about 1%, and pain relief if reported in up to 80% of patients [51].

The Frey and Beger procedures were developed with the underlying notion that the head of the pancreas functions like a pacemaker for pain in CP [51,53]. The Frey procedure consists of excising the anterior head of the pancreas, including the major and minor pancreatic ducts and the duct of Santorini, with preservation of the posterior pancreatic head and the pancreatic neck [51]. Additionally, the main pancreatic duct is opened, and a Roux-en-Y limb is brought up to complete a pancreaticojejunostomy [51]. Studies have shown that post-operative morbidity in the Frey procedure ranges from 7.5% to 39%, with mortality rates ranging from 0% and 2.4% [51,54-56]. A recent study of patients who underwent a Frey procedure found that over 90% of patients had full alleviation of pain [57].

Beger’s procedure is notorious for being one of the first procedures that consist of a pancreatic head resection while still being able to preserve the duodenum [51,58]. Beger’s procedure is also referred to as duodenum-preserving pancreatic head resection (DPPHR). This procedure is done by first preserving the posterior branch of the gastroduodenal artery, which supplies the duodenum, the intrapancreatic segment of the CBD, and the pancreaticoduodenal groove [51]. Next, there is resection of the neck and head of the pancreas, specifically at the area superimposing the portal vein and SMV, with a conscious effort to preserve pancreatic tissue present at the innermost portion of the duodenum [51,59]. A study on the outcomes of Beger’s procedure revealed that 91.7% of patients reported being pain-free after a median of 5.7 years [51,59]. Beger’s procedure has been found to be efficacious in patients with inflammatory masses at the head of the pancreas [51,59]. While Frey’s procedure also addresses disease associated with a head mass, the inability to rule out malignancy is an absolute contraindication for performing Frey’s procedure [60]. Beger’s procedure avoids major surgical resection of the CBD, duodenum, and portal vein and circumvents the need to restore bile flow, food passage, and portal blood flow, while still preserving the endocrine function of the pancreas [60]. Frey’s procedure is generally considered to be simpler than Beger’s; however, studies comparing both procedures have shown no differences in postoperative morbidity, pain relief, exocrine insufficiency, and quality of life [61].

**Figure 1:**
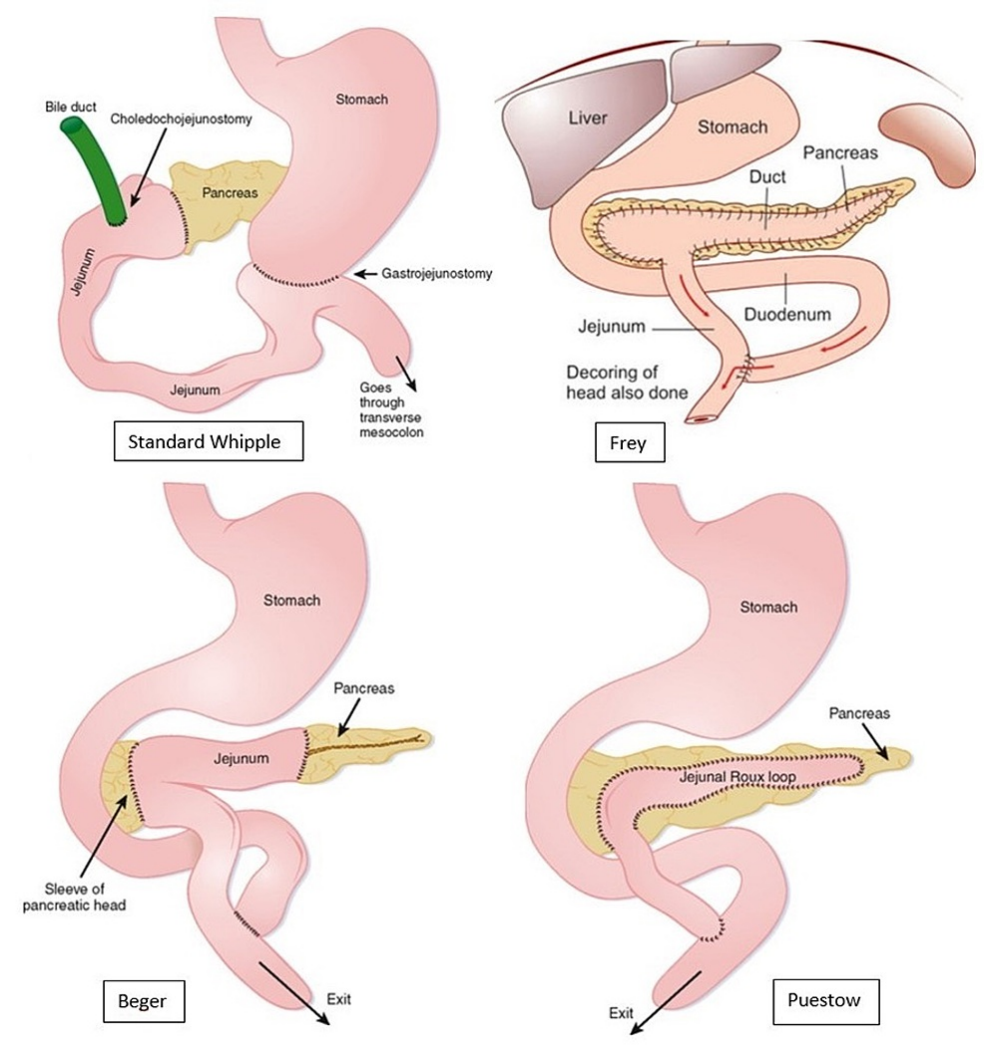
Depiction of the final gross anatomical changes associated with the Whipple, Frey, Beger, and Puestow procedures.

A distal pancreatectomy is indicated for those with distal pancreatic disease and simultaneous small duct diameter, as well as for those who have undergone a failed Puestow procedure, those with a pseudoaneurysm in the pancreatic tail, portal hypertension secondary to small duct disease, or suspicion of malignancy in the pancreatic tail [60]. This procedure is associated with the risk of symptomatic recurrence, with long-term relief achieved in only 60% of patients, and complete pancreatectomy needed in 13% of patients. Furthermore, approximately half of patients also develop exocrine and endocrine insufficiency [60]. Historically, the Whipple operation, or a pancreaticoduodenectomy, was the gold standard procedure for managing CP [51,62]. However, in recent times, the Whipple procedure is used almost exclusively for resectable pancreatic ductal adenocarcinomas, rather than for CP [62]. Although the Whipple operation was shown to be effective in the treatment of pain, there was evidence of significant long-term morbidity and complications [51]. A study conducted by Izbicki et al. showed that two years after surgery, the rate of in-hospital complication for patients who underwent the Whipple operation was 53.3%, compared with 19.4% in those who underwent the Frey procedure. Furthermore, global quality of life improved by 71% in those who underwent the Frey procedure, compared to 43% in those who underwent the Whipple procedure [63]. In a meta-analysis, it was determined that both the short- and long-term outcomes of the Berger and Frey procedures regarding the global quality of life were ominously better than the Whipple procedure [51,64]. A pictorial overview of the aforementioned surgical procedures is depicted in Figure 1 [65,66]. The last resort procedure is a total pancreatectomy, which is associated with severe morbidity secondary to brittle diabetes and lethal episodes of hypoglycemia [60].

## Conclusions

Pancreatitis is a complex pathology and its management is rooted in an intertwinement of both medical and surgical interventions. Our review explores and summarizes the various elements of pancreatitis including pancreatic embryology, anatomy, the pathophysiology of the disease, and its medical and surgical management. Knowledge of the possible course of disease along with knowledge of the different forms of management indicated for each potential consequence of pancreatitis is vital for halting disease progression, providing symptomatic relief, and reducing both morbidity and mortality.
